# Tolerance to surgically induced anisometropia—A systematic review

**DOI:** 10.1111/aos.17549

**Published:** 2025-06-28

**Authors:** Anne Guldhammer Skov, Morten la Cour, Birgitte Moldow, Lars Holm, Therese Krarup

**Affiliations:** ^1^ Department of Ophthalmology Copenhagen University Hospital – Rigshospitalet Copenhagen Denmark; ^2^ Faculty of Health Sciences University of Copenhagen Copenhagen Denmark

**Keywords:** Anisometropia, cataract, cataract surgery, monovision, presbyopia, refractive surgery, satisfaction

## Abstract

Surgically induced anisometropia is well tolerated by some individuals, while others experience binocular visual complaints. Surgically induced anisometropia is a common treatment for presbyopia and may also intentionally or non‐intentionally be the results after surgery for unilateral cataract with ametropia in the fellow eye. Research has shown significant variations in the tolerance of anisometropia. The aim of this systematic review was to examine the correlation between the degree of anisometropia, patient satisfaction and binocular visual complaints. We searched the databases: PubMed, EMBASE, Google Scholar, Cochrane and Web of Science for relevant reported studies. The search was supplemented with ongoing trials from www.clinicaltrials.gov. We searched for the following words: Anisometropia or monovision and satisfaction, aniseikonia, asthenopia, diplopia, headache, dizziness or fatigue. Our inclusion criteria encompassed all original studies, observational studies and randomized clinical trials that reported on clinical symptoms in patients with induced anisometropia. We included studies with induced anisometropia through cataract surgery or refractive surgery. Out of 1701 studies identified, 35 were included (*N*: 3186 patients): six randomized and 29 non‐randomized studies. Six studies compared patients with surgically induced anisometropia to distance corrected emmetropic patients. Follow‐up periods ranged from one to 32 months, with patient age from 22 to 96 years. Twenty‐two studies reported on surgically induced anisometropia after cataract surgery, 13 after corneal refractive surgery and reported a combined mean anisometropia of 1.57 diopters (D). Overall, patient satisfaction with surgically induced anisometropia was generally high and comparable with emmetropia in four of six comparative studies. Independence from reading glasses was higher in anisometropic patients, while independence from glasses for distance vision was higher in emmetropic patients. Asthenopia was generally reported by few patients, and when it was reported, it was generally mild. In conclusion, this systematic review and meta‐analysis of 35 studies involving 3186 patients suggests that surgically induced anisometropia just below 1 D is a viable approach to achieving high patient satisfaction with high degree of independence from reading glasses and minimal asthenopia. The majority of studies report high patient satisfaction and minimal incidence of asthenopia with no significant difference between anisometropic patients and distance‐corrected emmetropic patients.

## 1 INTRODUCTION

### 1.1 Rationale and objectives

Achieving spectacle‐free distance vision is possible with cataract and refractive surgery, but treating pseudophakic and age‐related presbyopia remains a challenge, as the available treatments are not yet optimal.

Monovision is the adjustment of one eye for distance vision and the fellow eye for near vision. It can be induced surgically either by cataract surgery or corneal surgery. Monovision is a well‐established strategy for treating presbyopia (Davidson et al., 2016) and has proven effective to overcome presbyopia and achieving spectacle independence for distance and near vision (Epstein & Gurgos, 2009; Greenstein & Pineda II, 2017; Wolffsohn & Davies, 2019). However, it also poses the risk of binocular visual complaints (Naeser et al., 2014).

Surgeons typically aim to induce anisometropia below 2–3 diopters (D) to minimize the risk of binocular visual complications (Berens & Bannon, 1963; Campos & Enoch, 1980; Katsumi et al., 1986; Ogle, 1950). However, studies have demonstrated significant variability in patient tolerance to anisometropia, and there is considerable variation in the literature regarding the recommended degree of intentionally induced anisometropia. Some experts suggest that monovision of 1.0 D is optimal for treating presbyopia, offering spectacle independence for both distance and intermediate vision with minimal binocular complaints (Naeser et al., 2014), while others advocate for monovision just under 3 D to achieve spectacle independence (Greenbaum, 2002).

The disadvantage of the present general practice of aiming for anisometropia below 2–3 D is the lack of evidence, and it is unsuitable in cases of unilateral cataract with significant refractive error in the fellow eye. In such cases, most surgeons will recommend bilateral surgery solely to prevent anisometropia above 2–3 D, which exposes the eye with good vision to unnecessary surgical risks. Alternatively, if surgery is performed only on one eye, the surgeon may have to aim for a less ideal target refraction to minimize anisometropia. Preoperative testing for tolerance of anisometropia using contact lenses can help avoid unnecessary bilateral surgery (Davidson et al., 2016). However, this approach is time consuming, often poorly tolerated by older patients, and the results are uncertain in patients with significant cataract.

Given the permanent nature of surgically induced anisometropia, the development of a data‐driven guideline about the tolerated degree of anisometropia to individual patients is desirable.

The aim of this review was to compile and evaluate the current research on surgically induced anisometropia, focusing on its correlation with patient satisfaction and binocular visual complaints.

## 2 METHOD

### 2.1 Eligibility criteria

A systematic search of the published literature was conducted, which included all original studies, including observational studies of patient series, and randomized and non‐randomized controlled trials, that reported on patient satisfaction or the prevalence or incidence of clinical symptoms in patients with surgically induced anisometropia, either for presbyopia or unilateral surgery with significant ametropia in the fellow eye. We included studies that induced anisometropia either by cataract surgery or by corneal refractive surgery (Laser in situ keratomileusis (LASIK), Klex (Kerato‐lenticule extraction), laser epithelial keratomileusis (LASEK) or photorefractive keratectomy (PRK)). We excluded studies that induced anisometropia by corneal inlays or by conductive keratoplasty. Likewise, we excluded duplicate cohorts. Furthermore, we excluded studies involving patients with axial anisometropia, neurological diseases and ocular disorders, including amblyopia, tropia or phoria. Many studies were excluded during screening as they addressed patient satisfaction or asthenopia caused by factors unrelated to surgically induced anisometropia such as anisometropia via contact lenses, or asthenopia caused by neurological diseases, other systemic diseases or ocular diseases. We included publications written in English, Danish, French, German, Norwegian or Swedish. There was no limitation to the publication period.

### 2.2 Information sources

We searched the databases PubMed, EMBASE, Google Scholar, Cochrane databases and Web of Science for relevant studies. Words found as Emtree words were searched as such and as free words in EMBASE. The reference lists from the included publications were screened for eligible studies that were not identified during the primary search. In addition, we searched www.clinicaltrials.gov for relevant ongoing studies. Covidence (https://www.covidence.org/, n.d.) was used to manage the records identified. The last search date was May 2024.

### 2.3 Search strategy

The following search terms were used:

Search: ((((((Anisometropia) OR (“mono vision”)) OR (monovision)) OR (mono‐vision))) AND ((((((((satisfaction) OR (aniseikonia)) OR (asthenopia)) OR (diplopia)) OR (headache)) OR (dizziness)) OR (fatigue)))) Filters: Danish, English, French, German, Norwegian, Swedish.

### 2.4 Selection process

One author (AS) performed the literature search for eligibility records. The search yielded 1701 records, which were further screened for inclusion using title and abstract screening or full‐text review. Two authors (AS and TK) independently screened the articles for inclusion or exclusion with reasons. In case of disagreement, a third author (LH) screened the article for inclusion.

### 2.5 Data collection and risk of bias assessment

Study data were collected using a data table, encompassing details on study design, participant characteristics, surgical interventions, exclusion criteria, postoperative refraction, postoperative patient satisfaction, postoperative asthenopia and postoperative spectacle use. Whenever feasible, individual participant data were extracted. In studies that included a subset of relevant participants, only eligible participants were included. For studies comprising more than two groups, data were pooled into either an anisometropic or emmetropic group. When methodologically feasible, a mean anisometropia was computed. Four studies were excluded from the statistical analysis due to missing data on anisometropia (Ayoubi et al., 2010; Hillman & Hawkswell, 1985; Kramer et al., 1999; Stock et al., 2017).

The risk of bias in the individual comparative studies of spectacles use was assessed independently by two authors (AS and TK). The Newcastle‐Ottawa Assessment Scale (NOS) (Lo et al., 2014) for non‐randomized case control studies was used. The NOS assigns up to ten points in three domains: (1) selection of study groups, (2) comparability of groups and (3) ascertainment of exposure. The bias in each domain was reported as high (0–1 point), with some concern (2 points) or low (3+ points). An overall assessment was reported as very good (9–10 points), good (7–8 points), satisfactory (5–6 points) and unsatisfactory (0–4 points). Any disagreements between authors were resolved through discussion. Microsoft Excel 2016 was used for data collection.

### 2.6 Data synthesis and analysis

All statistical analysis was conducted using Review Manager (RevMan version 8.14.0, The Cochrane Collaboration) (The Cochrane Collaboration, n.d.). Statistical plots were performed in R Software for Statistical Computing version 4.4.1, using the ggplot2 and wCorr packages. Where applicable, studies were pooled for meta‐analysis and a risk ratio with 95% confidence intervals (CI) was calculated. Heterogeneity between studies was checked and reported using Chi^2^ and *I* ^2^.

Where a meta‐analysis was not applicable, we synthesized data via plots, either error bar plots with CI (binominal CI) or bubble plots when it was the only solution. For the synthesis of patient satisfaction as a function of anisometropia, we pooled the results of anisometropic and emmetropic patients as well as we pooled the results of ‘patient reported overall satisfaction’ and ‘patient reported satisfaction with visual function’ since we assume, that the answers to the two different ways of assessing patient satisfaction both may reflect the same information. For comparability, all patient satisfaction scales were converted to numerical values ranging from 0 to 10, with 10 representing the highest satisfaction level. The midpoint of the 0–10 scale (5) was defined as the neutral point. The categorical scales were either two‐step or three‐step scales. For the two‐step scales, ‘satisfaction’ was assigned the value 7.5 and ‘dissatisfaction’ was assigned the value 2.5. For the three‐step scales, ‘satisfaction’ was assigned the value 8.335, the neutral level was assigned the value 5 and ‘dissatisfaction’ was assigned the value 1.665. Numerical scales were converted by relevant subtraction and multiplication.

A weighted Pearson correlation was computed. For the synthesis of asthenopia as a function of anisometropia, we pooled the results of anisometropic and emmetropic patients, and we converted results to a 0–10 scale for comparability, where 10 represents the highest symptom score. A weighted Pearson correlation was computed. For the synthesis of spectacles independence, all results were converted to spectacles independence, as it was assumed that spectacles independence was the reciprocal of spectacles use. Studies providing a prevalence of spectacles use or independence were used to compute an error bar plot with CI.

### 2.7 Data items

The primary outcome of this systematic review was patient satisfaction and binocular visual complaints in patients with surgically induced anisometropia. We made our search based on these primary outcomes. Secondary outcomes were spectacles use and adverse events.

## 3 RESULTS

A total of 1701 studies were initially identified, which were reduced to 1050 after removal of duplicates. During the screening process, 891 studies were excluded, resulting in 159 eligible for full‐text review. Of these, an additional 124 studies were excluded, leading to a final inclusion of 35 studies in the systematic review (Abdelrazek Hafez & Helaly, 2019; Ayoubi et al., 2010; Barisić et al., 2010; Cao et al., 2012; Chen et al., 2007; Finkelman et al., 2009; Fu et al., 2018; Goldberg, 2001, 2003; Greenbaum, 2002; Handa et al., 2004; Hillman & Hawkswell, 1985; Ito et al., 2009; Jain et al., 2001; Kim et al., 2015; Kramer et al., 1999; Krarup et al., 2020; Labiris et al., 2015, 2017; Lan et al., 2023; Levinger et al., 2013; Lubiński et al., 2013; Mäkinen et al., 2019; Marques et al., 2009; Peng et al., 2018; Reilly et al., 2006; Rodov et al., 2019; Rutstein et al., 2015; Schallhorn et al., 2017; Stock et al., 2017; Verdoorn, 2017; Wilkins et al., 2013; Xun et al., 2020; Zettl et al., 2014; Zhang et al., 2015).

### 3.1 Study selection

**FIGURE 1:**
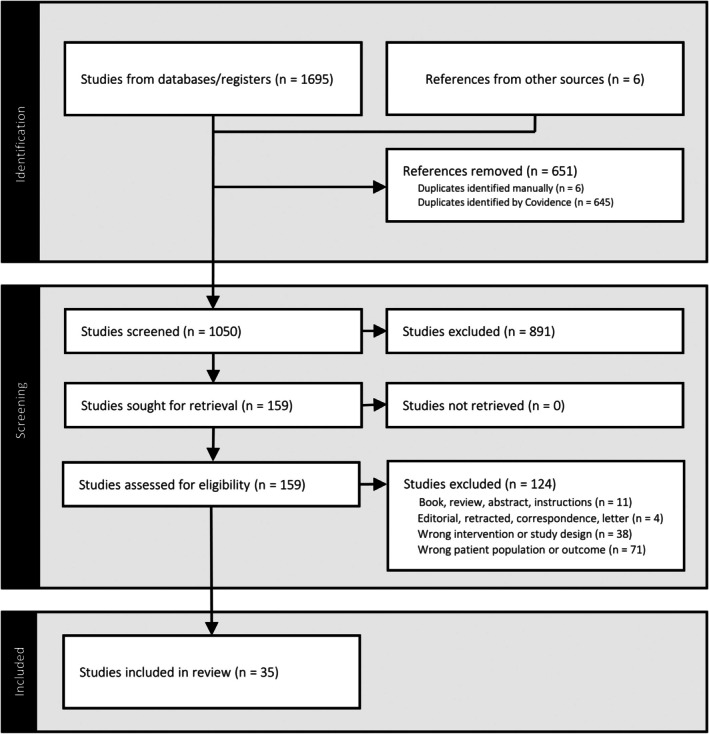
PRISMA flow chart illustrating the article search.

A flow chart illustrating the article search process is presented in Figure 1.

### 3.2 Risk of bias

Risk of bias was assessed in terms of specific domains: Selection of study groups was associated with a low risk of bias across all studies, comparability of groups had a high risk of bias in all studies and ascertainment of exposure was of some concern in two studies and with a high risk of bias in one study. The overall assessment was satisfactory for the two studies by Goldberg (2001, 2003) and good for the study by Rodov et al. (2019).

### 3.3 Study characteristics

A total of 3186 patients were enrolled, 56% of whom were women (*N*: 1682, based on the studies that reported gender distribution). The patients' ages ranged from 22 to 96 years. The follow‐up period varied between one and 32 months.

Six of the studies were randomized, while the remaining were non‐randomized patient series or clinical studies, consisting of 20 retrospective and nine prospective. Six studies compared anisometropic patients to patients with distance‐corrected emmetropia, and none of these studies were randomized.

Characteristics of the 29 non‐comparative studies of anisometropic patients are summarized in Table 1, while those of the six comparative studies of anisometropic patients and distance‐corrected emmetropic patients are summarized in Table 2.

**TABLE 1:** Baseline characteristics of the 29 non‐comparative studies of anisometropic patients.

Author (pub. year)	Study design	Patients, *N*	Age, mean ± SD, years	Method	Eyes operated
Abdelrazek Hafez and Helaly (2019)	Retrospective	30	56.3 ± 5.5	Cataract surgery	Both
Chen et al. (2007)	Retrospective	20	50–80	Cataract surgery	Both
Finkelman et al. (2009)	Prospective	26	67.4	Cataract surgery	1
Greenbaum (2002)	Prospective	140	68.4	Cataract surgery	Both
Handa et al. (2004)	Prospective	20	60	Cataract surgery	1
Hillman and Hawkswell (1985)	Retrospective	50	NR	Cataract surgery	1
Ito et al. (2009)	Retrospective	96	67.9 ± 8.2	Cataract surgery	Both
Kim et al. (2015)	Randomized	59	69.05	Cataract surgery	Both
Kramer et al. (1999)	Prospective	87	36–90	Cataract surgery	1 or both
Labiris et al. (2015)	Randomized	38	59.5 ± 10.4	Cataract surgery	Both
Labiris et al. (2017)	Randomized	62	60.1 ± 9.2	Cataract surgery	Both
Lan et al. (2023)	Prospective	43	66.0 ± 6.4	Cataract surgery	Both
Lubiński et al., 2013	Retrospective	20	62.15 ± 7.22	Cataract surgery	Both
Marques et al. (2009)	Prospective	38	64.4 ± 12.99	Cataract surgery	Both
Rutstein et al. (2015)	Prospektive	17	54–75	Cataract surgery	1
Stock et al. (2017)	Prospective	29	≤85	Cataract surgery	Both
Wilkins et al. (2013)	Randomized	93	68.7 ± 12.0	Cataract surgery	Both
Xun et al. (2020)	Randomized	46	66.88 ± 11.20	Cataract surgery	Both
Zettl et al. (2014)	Retrospective	30	73 (median)	Cataract surgery	Both
Zhang et al. (2015)	Retrospective	60	72 ± 6.5	Cataract surgery	Both
Ayoubi et al. (2010)	Retrospective	32	55 ± 4.3	LASIK	1
Barisić et al. (2010)	Randomized	50	47 ± 1.7	LASIK	Both
Cao et al. (2012)	Retrospective	40	44.5	LASIK	Both
Fu et al. (2018)	Retrospective	30	45.53 ± 3.20	SMILE	Both
Levinger et al. (2013)	Prospective	40	48.3 ± 5.7	LASIK or LASEK	1 or both
Peng et al. (2018)	Retrospective	294	52.5 ± 4.5	LASIK	1 or both
Reilly et al. (2006)	Retrospective	82	>40	LASIK	1 or both
Schallhorn et al. (2017)	Retrospective	608	45–60	LASIK	Both
Verdoorn (2017)	Retrospective	15	56	LASIK	1 or both

Abbreviations: LASEK, laser epithelial keratomileusis; LASIK, laser in situ keratomileusis; *N*, patient number; NR, not reported; SD, standard deviation; SMILE, Small Incision Lenticule Extraction.

**TABLE 2:** Baseline characteristics of the six comparative studies of anisometropic patients and distance‐corrected emmetropic patients.

Author (pub. year)	Study design	Anisometropic patients, *N*	Emmetropic patients, *N*	Age, anisometropic patients, mean ± SD, years	Age, anisometropic patients, mean ± SD, years	Method	Eyes operated
Krarup et al. (2020)	Retrospective	123	17	69 ± 7.87	67 ± 6.15	Cataract surgery	1 or both
Rodov et al. (2019)	Retrospective	50	50	66.79 ± 9.86	72.18 ± 7.87	Cataract surgery	Both
Goldberg (2001)	Retrospective	114	119	40‐63	40–67	LASIK	Both
Goldberg (2003)	Retrospective	137	251	>40	>40	LASIK	Both
Jain et al. (2001)	Retrospective	42	35	49.7 ± 3.9	48.4 ± 2.8	LASIK or PRK	1 or both
Mäkinen et al. (2019)	Retrospective	44	9	22‐65	22–65	LASIK	1 or both

Abbreviations: LASIK, laser in situ keratomileusis; *N*, patient number; NR, not reported; PRK, photorefractive keratectomy; SD, standard deviation.

Most of the included studies were conducted with intentions other than investigating the tolerable degree of anisometropia. We extracted relevant data for our purpose, knowing that this may not be optimal, but it was the best available approach to obtain information for this review.

### 3.4 Results of individual studies

Six of the 35 included studies compared patients with surgically induced anisometropia to patients with surgically induced distance corrected emmetropia (Goldberg, 2001, 2003; Jain et al., 2001; Krarup et al., 2020; Mäkinen et al., 2019; Rodov et al., 2019) (*N*: 991). We defined the distance‐corrected emmetropic patients as a control group for the anisometropic patients, and these six studies were used to compare relevant endpoints to investigate the tolerance to anisometropia. In this comparison, we examined the postoperative outcomes between the two groups, rather than the changes from preoperative to postoperative. In the non‐comparative studies, relevant postoperative endpoints were included in the data analysis in cases where it was found relevant to elucidate an outcome, although there was no associated control group.

### 3.5 Patient selection and monovision design

All patients in the included studies were informed about the monovision design and were in part selected because of their desire for possible spectacles independence after the surgery. The majority of the included studies reported their exclusion criteria, with the most prominent being corneal astigmatism >1 D, ocular pathology that could affect postoperative visual acuity, amblyopia, strabismus and previous ocular surgery.

The monovision protocols varied across the studies. Some induced conventional monovision (correction of the dominant eye for distance vision), while others induced crossed monovision (correction of the non‐dominant eye for distance vision). The preoperative refraction differed as some studies included emmetropic patients, some included myopic patients, some included hypermetropic patients and some included a mix. Some studies utilized contact lens trials for all or some of the patients (Abdelrazek Hafez & Helaly, 2019; Ayoubi et al., 2010; Jain et al., 2001; Peng et al., 2018; Reilly et al., 2006; Schallhorn et al., 2017), whereas others did not. Surgical approaches also differed, with some studies performing unilateral surgery and others bilateral interventions. In this systematic review, we did not delve into these methodological variations, as our primary objective was to assess the tolerated degree of surgically induced anisometropia, independent of these specific factors.

Twenty‐two studies induced anisometropia via cataract surgery (Abdelrazek Hafez & Helaly, 2019; Chen et al., 2007; Finkelman et al., 2009; Greenbaum, 2002; Handa et al., 2004; Hillman & Hawkswell, 1985; Ito et al., 2009; Kim et al., 2015; Kramer et al., 1999; Krarup et al., 2020; Labiris et al., 2015, 2017; Lan et al., 2023; Lubiński et al., 2013; Marques et al., 2009; Rodov et al., 2019; Rutstein et al., 2015; Stock et al., 2017; Wilkins et al., 2013; Xun et al., 2020; Zettl et al., 2014; Zhang et al., 2015). All studies, except for one (Greenbaum, 2002), were classified as clinically significant cataract, as it is assumed that the indication for surgery was significant cataract when no indication was documented, and the patient age was above 50 years. The remaining 13 studies induced anisometropia via corneal refractive surgery (Ayoubi et al., 2010; Barisić et al., 2010; Cao et al., 2012; Fu et al., 2018; Goldberg, 2001, 2003; Jain et al., 2001; Levinger et al., 2013; Mäkinen et al., 2019; Peng et al., 2018; Reilly et al., 2006; Schallhorn et al., 2017; Verdoorn, 2017).

Overall, the mean anisometropia induced in patients with anisometropia based on studies providing specific estimates was 1.57 ± 0.88 D (*N*: 1343), with a maximum mean of 3.84 ± 0.58 (Rutstein et al., 2015). Seventeen studies induced a mean anisometropia below 2 D with a mean anisometropia of 1.45 D (Abdelrazek Hafez & Helaly, 2019; Cao et al., 2012; Finkelman et al., 2009; Jain et al., 2001; Kim et al., 2015; Krarup et al., 2020; Labiris et al., 2015, 2017; Levinger et al., 2013; Reilly et al., 2006; Rodov et al., 2019; Schallhorn et al., 2017; Verdoorn, 2017; Wilkins et al., 2013; Xun et al., 2020; Zettl et al., 2014; Zhang et al., 2015) (*N*: 1444). Five studies induced mean anisometropia above 2 D with a mean anisometropia of 2.46 ± 0.64 D (Handa et al., 2004; Ito et al., 2009; Lubiński et al., 2013; Marques et al., 2009; Rutstein et al., 2015) (*N*: 191).

In the six comparative studies, the mean anisometropia in emmetropic patients was below 1 D in all studies; about 0 D in four studies (Goldberg, 2001, 2003; Mäkinen et al., 2019; Rodov et al., 2019) and 0.5 in one study (Krarup et al., 2020).

### 3.6 Patient satisfaction

Thirty‐one studies reported results concerning patient satisfaction. Patient satisfaction was evaluated using various methods across the studies, ranging from validated questionnaires to data extracted from patients' medical records. All types of information sources were considered acceptable for inclusion in this review.

Overall, patient satisfaction was recorded as ‘patient reported overall satisfaction’ (Abdelrazek Hafez & Helaly, 2019; Ayoubi et al., 2010; Barisić et al., 2010; Finkelman et al., 2009; Fu et al., 2018; Goldberg, 2001, 2003; Greenbaum, 2002; Handa et al., 2004; Ito et al., 2009; Jain et al., 2001; Lan et al., 2023; Levinger et al., 2013; Reilly et al., 2006; Xun et al., 2020; Zhang et al., 2015) (*N*: 988 anisometropic/405 emmetropic) or ‘patient reported satisfaction with visual function’ (Chen et al., 2007; Kim et al., 2015; Krarup et al., 2020; Labiris et al., 2015, 2017; Lubiński et al., 2013; Mäkinen et al., 2019; Marques et al., 2009; Peng et al., 2018; Rodov et al., 2019; Schallhorn et al., 2017; Stock et al., 2017; Verdoorn, 2017; Wilkins et al., 2013; Zettl et al., 2014; *N*: 1523 anisometropic/76 emmetropic), either via a questionnaire or on a categoric or numeric scale.

**FIGURE 2:**
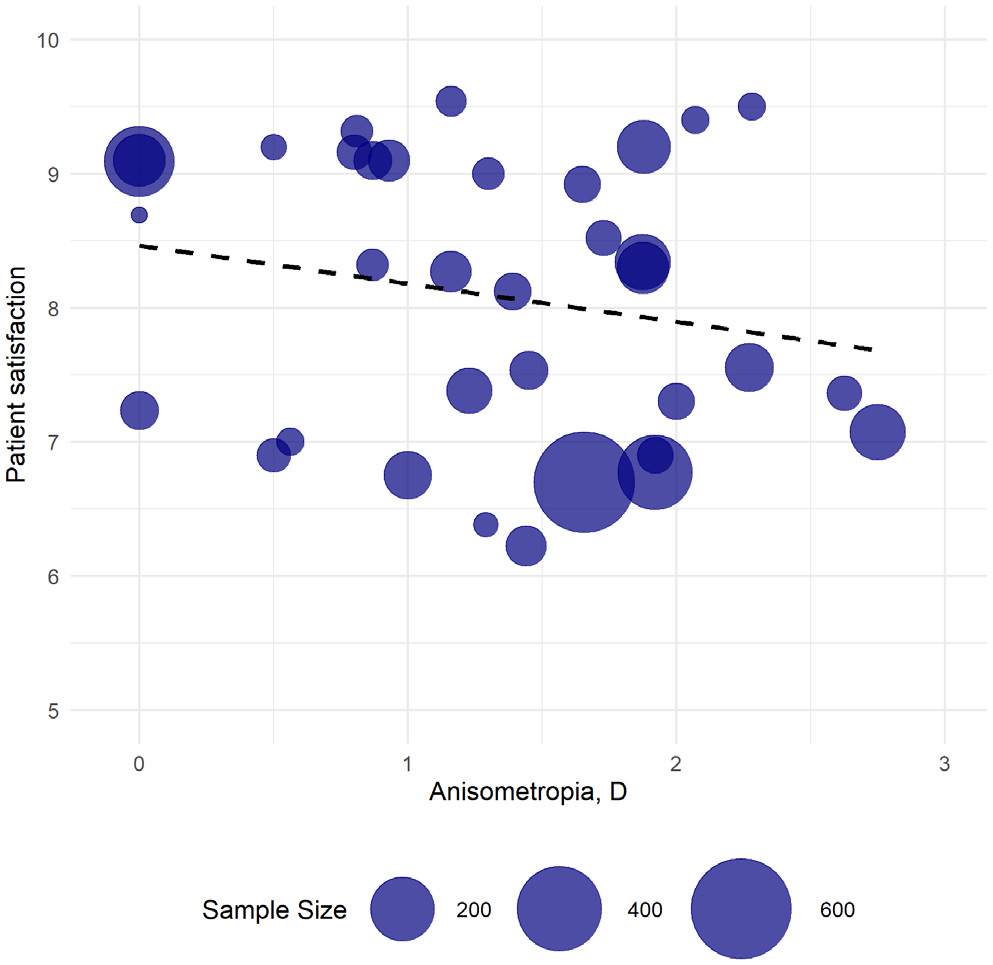
Bubble plot of patient satisfaction as a function of anisometropia with weighted correlation (−0.467). *X*‐axis values represent anisometropia (diopters (D)). *Y*‐axis represents patient satisfaction on a 0–10 satisfaction scale, where 10 represents highest satisfaction. Bubble size illustrates sample size.

Figure 2 illustrates the postoperative patient satisfaction as a function of anisometropia. Anisometropia ranged from 0 to 3 D, while patient satisfaction score ranged from 6.23 to 9.54 on a 0 to 10 scale. A slight negative correlation was observed between the degree of anisometropia and patient satisfaction, with a correlation of −0.467. In a subgroup analysis of anisometropia of 1 D or less, patient satisfaction remained similar, with a correlation of −0.362.

In the comparative studies of anisometropic patients and distance‐corrected emmetropic patients, there was no clear correlation between patient satisfaction and degree of anisometropia. In four out of six studies, there was no significant difference in patient satisfaction between the two groups (Goldberg, 2001; Jain et al., 2001; Krarup et al., 2020; Mäkinen et al., 2019) (*N*: 323 anisometropic/180 emmetropic). One study reported a statistically significant difference, with emmetropic patients being more satisfied than anisometropic patients (16) (*N*: 137 anisometropic/251 emmetropic), while the sixth study reported a statistically significant difference in near‐vision satisfaction, with anisometropic patients being more satisfied than emmetropic patients, but no difference in distance vision satisfaction (*p* < 0.0001) (Rodov et al., 2019) (*N*: 50 anisometropic/50 emmetropic).

### 3.7 Binocular visual complaints

**FIGURE 3:**
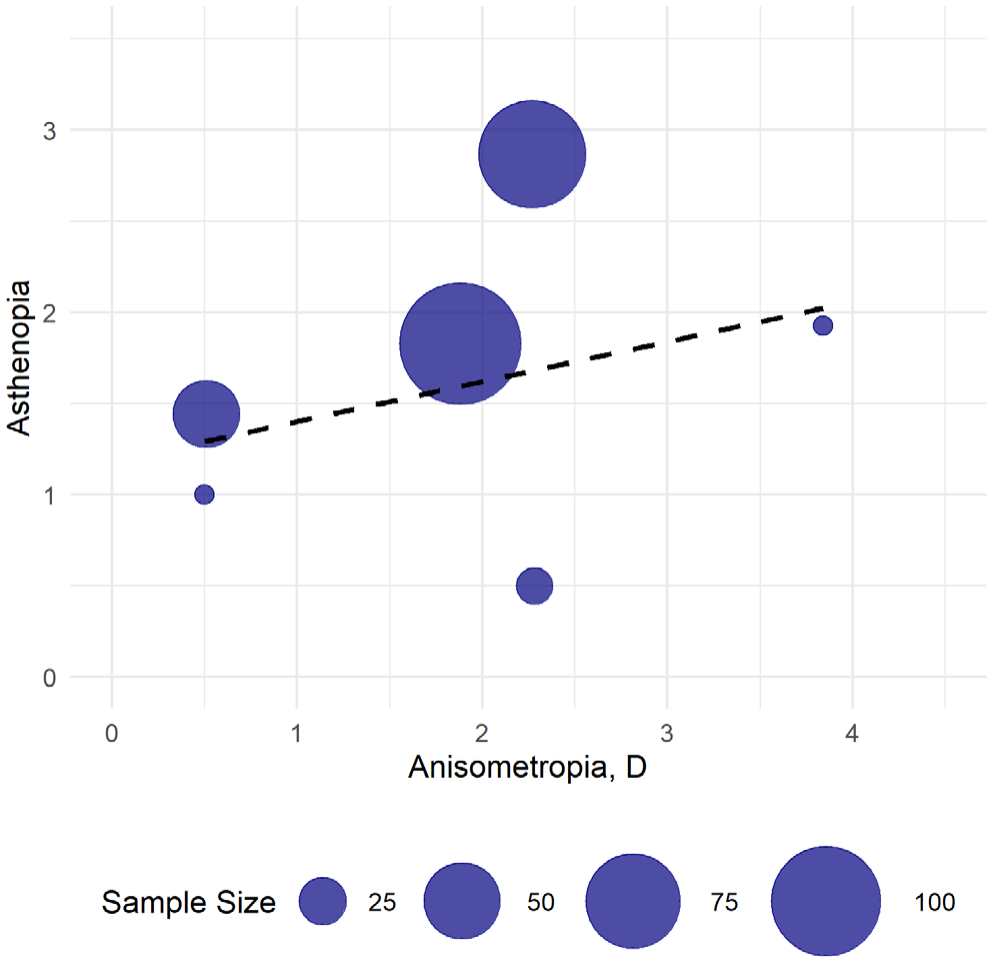
Bubble plot of asthenopia as a function of anisometropia with weighted correlation (0.426). *X*‐axis values represent anisometropia (diopters (D)). Y‐axis represent asthenopia on a 0–10 scale, where 10 represent highest symptom score. Bubble size illustrates sample size.

Binocular visual complaints were defined as asthenopia, comprising clinical aniseikonia, diplopia, headache, dizziness and fatigue. Seven studies reported postoperative asthenopia (Cao et al., 2012; Handa et al., 2004; Hillman & Hawkswell, 1985; Ito et al., 2009; Kramer et al., 1999; Krarup et al., 2020; Rutstein et al., 2015) (*N*: 433 anisometropic/17 emmetropic). Figure 3 illustrated the bubble plot of asthenopia as a function of anisometropia. In general, asthenopia was reported by few patients, and when it was reported by the patients, it was reported with a low score reflecting few symptoms. The maximum mean asthenopia score was 2.9 on a 0 to 10 score. There was a slightly positive correlation between higher degree of anisometropia and more asthenopia, with a correlation of 0.426.

Results from Kramer et al. (1999) were not synthesized due to methodological differences. However, they reported, as the only study, a high proportion of patients with asthenopia, noting that 42% of all pseudophakic patients experienced asthenopia.

In the comparative studies, Krarup et al. (2020) were the only study to report on asthenopia in the two groups. They reported on asthenopia via the Convergence Insufficiency Symptom Survey (CISS), finding no statistically significant difference between the groups (CISS score: 11 for anisometropic patients/6 for emmetropic patients).

### 3.8 Anisometropia and spectacles use

**FIGURE 4:**
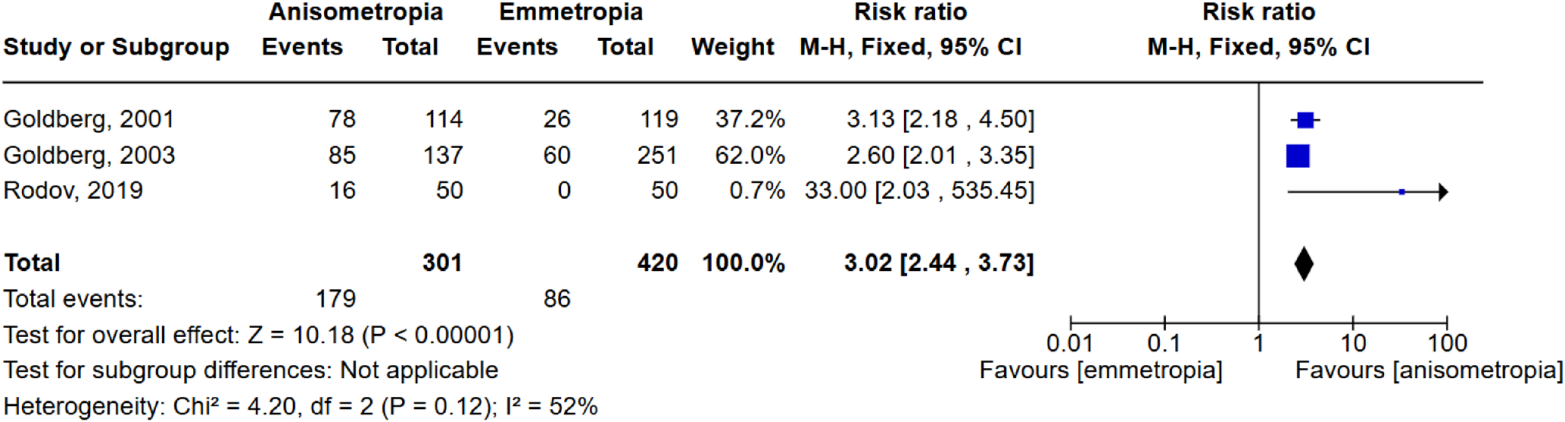
Meta‐analysis and forest plot of independence from reading glasses. CI, confidence interval.

**FIGURE 5:**
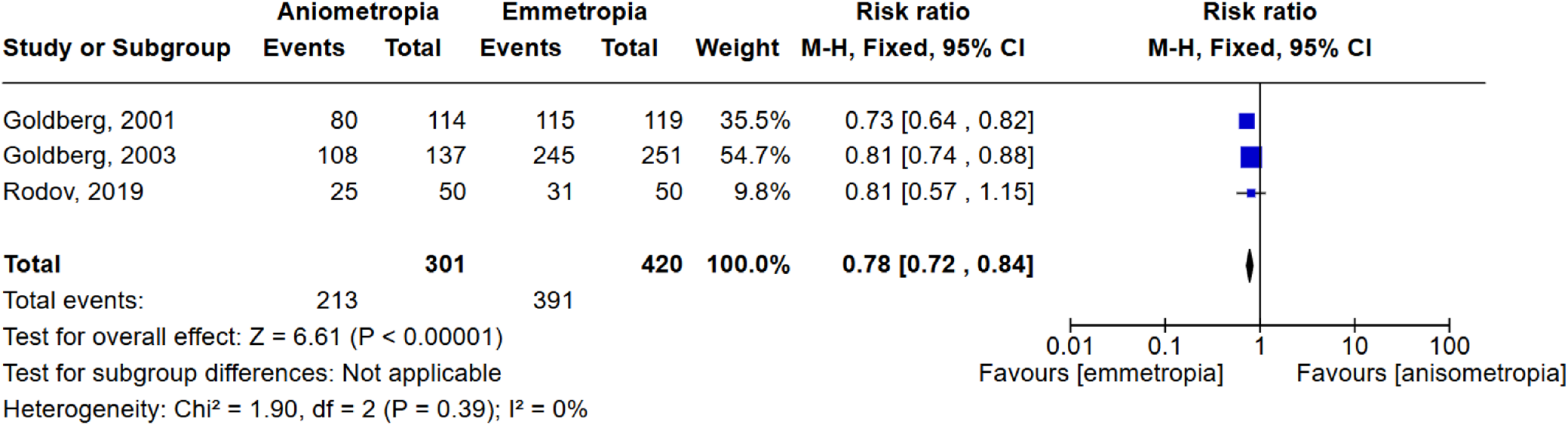
Meta‐analysis and forest plot of independence from glasses for distance vision. CI, confidence interval.

Twenty studies reported the outcome of spectacles usage (Abdelrazek Hafez & Helaly, 2019; Barisić et al., 2010; Chen et al., 2007; Finkelman et al., 2009; Fu et al., 2018; Goldberg, 2001, 2003; Greenbaum, 2002; Ito et al., 2009; Kim et al., 2015; Labiris et al., 2015; Lan et al., 2023; Levinger et al., 2013; Lubiński et al., 2013; Rodov et al., 2019; Rutstein et al., 2015; Wilkins et al., 2013; Xun et al., 2020; Zettl et al., 2014; Zhang et al., 2015) (*N*: 1139 anisometropic/420 emmetropic). Three were comparative of anisometropic and distance‐corrected emmetropic patients (*N*: 301 anisometropic/420 emmetropic). Figures 4 and 5 illustrate the risk ratio of independence from reading glasses and glasses for distance vision, respectively, in these three comparative studies. The pooled estimate for reading glasses was a relative risk of 3.02 (95% CI 2.44 to 3.73), favouring the anisometropic patients. The difference was statistically significant (*p* < 0.00001) with a moderate heterogeneity (*I* ^2^ = 52%). The pooled estimate for glasses for distance vision was a relative risk of 0.78 (95% CI 0.72 to 0.84), favouring the emmetropic patients. The difference was statistically significant (p < 0.00001) with a low heterogeneity (*I* ^2^ = 0%).

**FIGURE 6:**
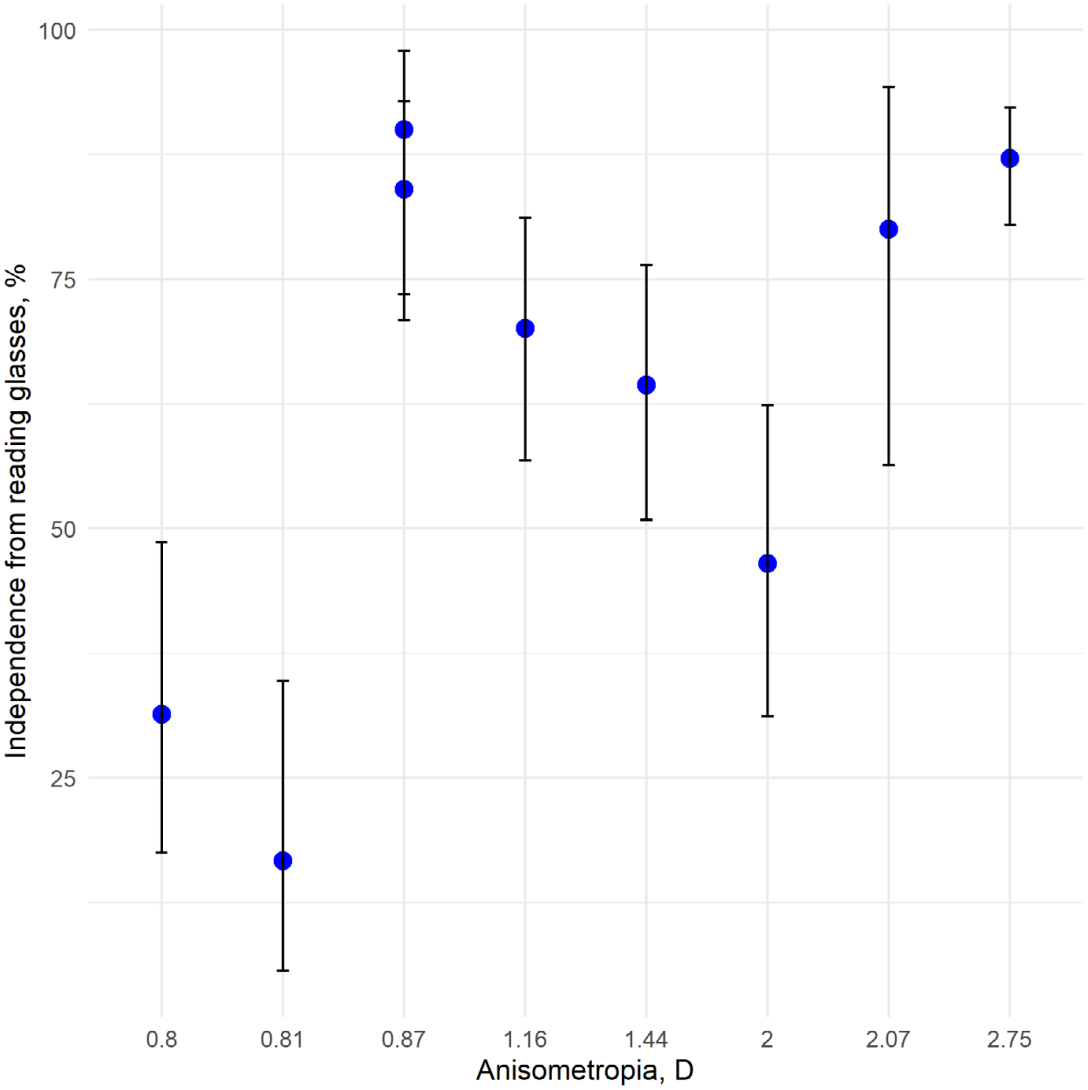
Error bar plot of independence from reading glasses. The mean prevalences are represented by dots, and the vertical error bars represent the 95% confidence intervals of the prevalences. *X*‐axis represent values of anisometropia (diopters (D)) for the included studies. Y‐axis values represent prevalence of independence from reading glasses (%).

**FIGURE 7:**
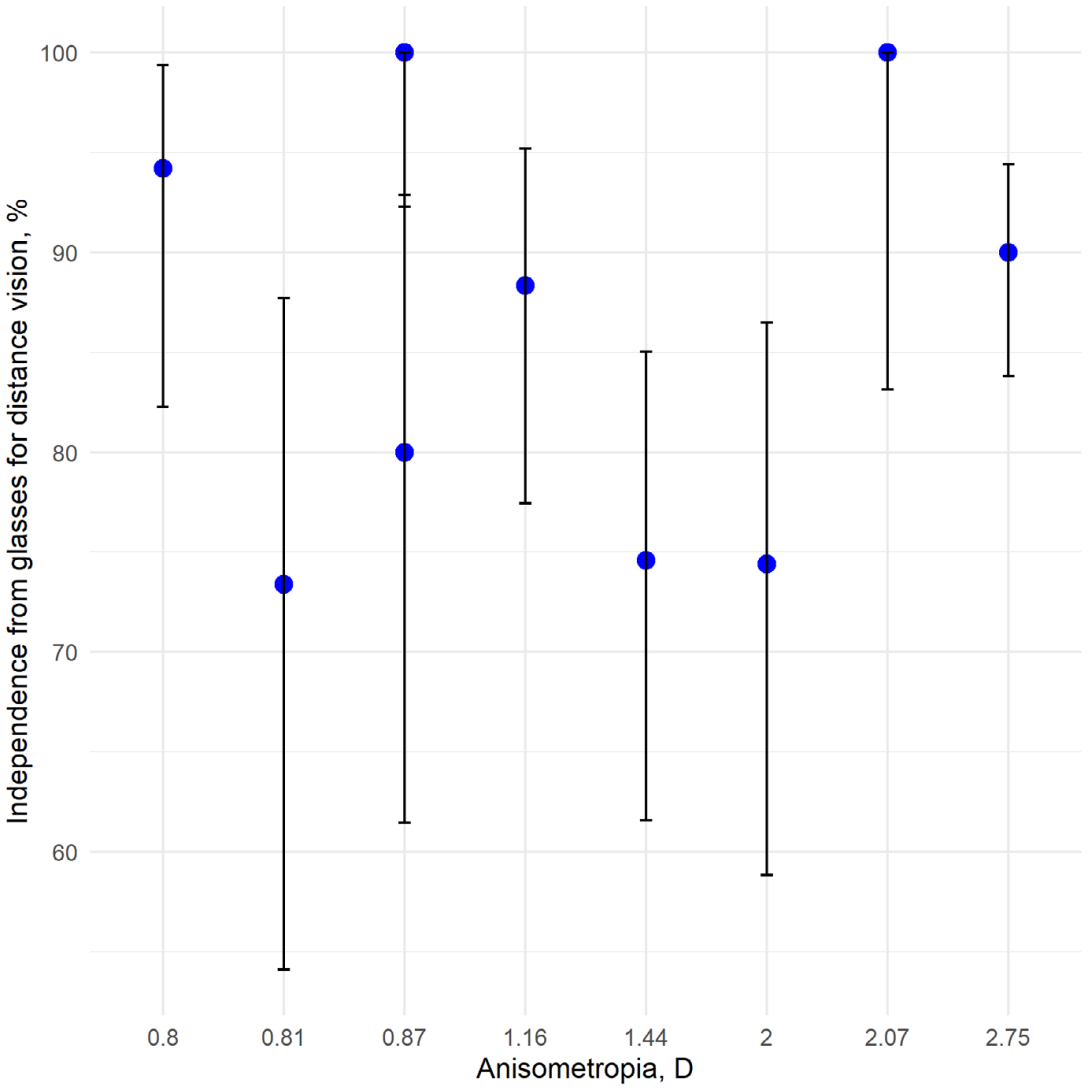
Error bar plot of independence from glasses for distance vision. The mean prevalences are represented by dots, and the vertical error bars represent the 95% confidence intervals of the prevalences. The *X*‐axis represents values of anisometropia (diopters (D)) for the included studies. The *Y*‐axis values represent prevalence of independence from glasses for distance vision (%).

Results of spectacles independence for the non‐comparative studies were synthesized. Figures 6 and 7 illustrates the results of independence from reading glasses and glasses for distance vision, respectively. The mean prevalence of independence from reading glasses ranges from 16.7% to 90%. Our analysis revealed a positive correlation between increased anisometropia and reduced need for reading glasses. The finding was insignificant. Lan et al. (2023) is an outlier with a high prevalence of patients using reading glasses.

The independence from spectacles for distance vision ranges from 73.4% to 100%. We found no correlation between the degree of anisometropia and independence from glasses for distance vision.

### 3.9 Systemic adverse events

None of the 35 included studies reported any systemic adverse events.

## 4 DISCUSSION

This systematic review of 35 studies, involving a total of 3186 patients, suggests that surgically induced anisometropia just below 1 D following cataract and refractive surgery is a viable approach to achieve high patient satisfaction and relative independence from reading glasses without inflicting asthenopia on the patients.

The mean surgically induced anisometropia was 1.57 ± 0.88 D; 17 studies induced anisometropia below 2 D (*N*: 1444) and five induced anisometropia above 2 D (*N*: 191).

Like previous studies, we found a statistically significant less frequent use of reading glasses in patients with anisometropia (mean anisometropia of 1.25 to 2.50 D) compared with distance‐corrected emmetropic patients, which was supported by the synthesis of anisometropic patients from the non‐comparative studies (non‐comparative meaning that there was neither an external nor a pre‐ to postoperative comparison). Regarding glasses for distance vision, we found a statistically significant less frequent use in patients with distance‐corrected emmetropia compared with anisometropic patients. However, we could not recover a trend toward less frequent use of glasses for distance with lower degree of anisometropia in the synthesis of results from anisometropic patients from the non‐comparative studies. With this, it seems that surgically induced anisometropia just less than 1 D is preferred as higher degree of anisometropia could not be shown to give greater independence from reading glasses in our data, and spectacle independence from glasses for distance favours low degree of anisometropia.

Patient satisfaction with surgically induced anisometropia after cataract and refractive surgery was generally high and comparable with distance‐corrected emmetropic patients in four of six comparative studies. We found a weak negative correlation of −0.467 between higher degree of anisometropia and patient satisfaction. It seems that patient satisfaction remains similar with anisometropia just below 1 D compared to patients with higher degree of anisometropia. Likewise, studies using contact lens trials did not report higher patient satisfaction, making the correlation between time consumption, risk of complications from contact lens wear, discomfort from contact lens use and the effect of the trials unclear. However, the results of patient satisfaction should be interpreted with caution, as the evidence level of assessment of patient satisfaction was low to moderate due to methodological heterogeneity among the studies.

The potential risk for binocular visual complaints, defined as asthenopia in this systematic review, following surgically induced anisometropia, is challenging to assess. It seems that few patients with surgically induced anisometropia report asthenopia, and that when they do, the symptoms are generally mild. A tendency toward a positive correlation between increased anisometropia and a higher prevalence of asthenopia was observed. However, this association could not be found in the results by Krarup et al. (2020), the only comparative study to report on asthenopia in anisometropic and distance‐corrected emmetropic patients. In their study, no statistically significant differences in asthenopia were found between the two groups, as assessed by the Convergence Insufficiency Symptom Survey (CISS). Conversely, the study by Kramer et al. (1999) reported a notably higher prevalence of asthenopia compared to the other studies, with 42% of pseudophakic patients experiencing asthenopia. Therefore, this systematic review does not provide a definitive answer regarding the prevalence of asthenopia in anisometropic and distance‐corrected emmetropic patients. The observed overall low prevalence of asthenopia may be accurate or could reflect the population studied, which was characterized by patients with relatively low degrees of anisometropia. However, the reported rate of asthenopia may be underestimated due to the variability in the assessment methods and the lack of standardization in reporting. Unpublished data from Krarup et al. (2020) support the high prevalence of asthenopia reported by Kramer et al., as an analysis of the raw data revealed that 90% of anisometropic patients and 94% of emmetropic patients had a CISS score greater than 0, indicating some degree of asthenopia.

In conclusion, this systematic review and meta‐analysis of 35 studies involving 3186 patients suggests that surgically induced anisometropia just below 1 D is a viable approach to achieving high patient satisfaction with a high degree of independence from reading glasses and minimal asthenopia. The majority of studies report high patient satisfaction and minimal incidence of asthenopia with no significant difference between anisometropic patients and distance‐corrected emmetropic patients. Future research, particularly randomized controlled trials utilizing validated questionnaires, along with a consensus‐driven standardization of patient satisfaction and side‐effect evaluation, is warranted to provide more definite insights into the effects and side effects of surgically induced anisometropia following cataract and refractive surgery.

## REGISTRATION AND PROTOCOL

For this systematic review, a protocol was prospectively registered at PROSPERO, International prospective register of systematic review (https://www.crd.york.ac.uk/prospero/). Registration no.: CRD42023451707. The Preferred Reporting Items for Systematic Reviews and Meta‐Analyses (PRISMA) (Page et al., 2021) statement checklist has been used.
